# Current Approaches to Desensitization in Solid Organ Transplantation

**DOI:** 10.3389/fimmu.2021.686271

**Published:** 2021-05-11

**Authors:** Carrie Schinstock, Anat Tambur, Mark Stegall

**Affiliations:** ^1^ William J. von Liebig Transplant Center, Mayo Clinic, Rochester, MN, United States; ^2^ Department of Surgery, Northwestern University, Chicago, IL, United States

**Keywords:** desensitization, sensitization, kidney transplantation, donor specific antibody (DSA), crossmatch, single antigen bead assays (SAB), antibody mediated rejection, kidney paired donation

## Abstract

Major advancements in the development of HLA antibody detection techniques and our understanding of the outcomes of solid organ transplant in the context of HLA antibody have occurred since the relevance of sensitization was first recognized nearly 50 years ago. Additionally, kidney paired donation programs (KPD) have become widespread, deceased donor allocation policies have changed, and several new therapeutic options have become available with promise to reduce HLA antibody. In this overview we aim to provide thoughtful guidance about when desensitization in kidney transplantation should be considered taking into account the outcomes of HLA incompatible transplantation. Novel therapeutics, desensitization endpoints, and strategies for future study will also be discussed. While most of our understanding about desensitization comes from studying kidney transplant candidates and recipients, many of the concepts discussed can be easily applied to desensitization in all of solid organ transplantation.

## Introduction

Our understanding and perspectives surrounding desensitization in kidney transplant candidates has advanced in the last 2 decades as HLA antibody detection and measurement techniques have improved, leading to better clarity about who is likely to benefit from aggressive desensitization approaches. In this overview we aim to provide guidance about when desensitization should be considered. Novel therapeutics, desensitization endpoints, and strategies for future study will also be discussed. While most of our understanding about desensitization comes from studying kidney transplant candidates and recipients, many of the concepts discussed can be easily applied to desensitization in all of solid organ transplantation.

## What Is Sensitization and How Is It measured?

The significance of alloantibody was made evident early in transplantation when Dr. Terasaki observed the correlation between early allograft loss and *in vitro* lysing of donor cells after application of recipient serum (e.g. a positive crossmatch) (1). Other notable observations from this landmark study were that patients with prior exposure to alloimmune sensitization including females with a history of pregnancy or recipients of prior transplants, had a higher incidence of immediate failure (1). At that time it was believed that alloantibody was only relevant in the early post-transplant period, but eventually it was acknowledged that donor-specific alloantibody (DSA) towards HLA (human leukocyte antigen) was a major contributor to long term allograft loss through chronic active antibody mediated rejection (2), and thus DSA to HLA is avoided if possible. Recently there has been interest in better understanding the contribution of non-HLA antibody to rejection and graft loss. This is an evolving area of study with many unanswered questions. It also remains unclear whether the presence of non-HLA antibody leads to reduced access to transplantation, thus this review will be focused on sensitization in the context of HLA antibody only.

Detecting HLA antibody in a transplant candidate’s serum and measuring sensitization to determine which HLA antigens must be avoided at transplant is a critical first step to avoid DSA. Historically, cell-based panel reactive antibody (PRA) testing was performed to assess sensitization. Attempts were made to use cell panels representing the donor pool in order to estimate the proportion of the population to which the candidate would likely have preexisting DSA, however precise identification of individual antibody specificities was difficult. Techniques to detect and measure HLA-alloantibody have substantially improved. Currently sensitive single antigen bead (SAB) solid phase assays comprised of broad panels of fluorescent HLA-coated microbeads are available that allow for specific HLA alloantibody determination and semi-quantitative measurement. The results of these tests can be used to determine the calculated panel-reactive antibody (cPRA) and establish the breadth of sensitization to predict the probability of finding a donor against whom the recipient has no antibody (3). The cPRA ranges from 0-100% and can be easily calculated with a readily available online calculator that now provides the detailed cPRA (e.g. cPRA 99.555999%) (3). In the United States, the cPRA is used for determining deceased donor allocation priority.

Importantly, the cPRA is dependent upon which antigens are considered unacceptable by a specific transplant center based on the solid phase assay results. For example, a center with minimal risk tolerance for preformed DSA may exclude antigens when the corresponding MFI (mean fluorescence intensity) is very low (e.g. >500), while other centers with more tolerance for antibody mediated rejection (ABMR) risk may use a higher MFI cutoff.

Solid phase assays are almost universally used to measure sensitization clinically, but it is essential to understand that these tests have inherent limitations and do not measure immunologic memory. This is a critical point as the potential of immunologic memory response to the transplant organ has clinical relevance. Immunologic memory is defined as the robust response from the immune system when a foreign antigen that was previously encountered by the immune system is reintroduced, leading to reactivation of memory T and B cells. Immunologic memory can occur as a result of sensitizing events such as pregnancy, prior kidney transplant, blood transfusion, and implants such as homografts. In other words, ABMR remains possible, albeit at low risk, even when solid phase and lymphocyte crossmatch testing using current serum samples are completely negative. Therefore, the results of solid phase assays must be interpreted in the context of a patient’s sensitization history and historic results if available. A variety of techniques are available to study antigen-specific B-cell responses in the research setting, but none have been validated to be used routinely in the clinical setting (4).

Terms such as *highly sensitized* are routinely used in the field of transplantation without a universal meaning. Historically even patients with a cPRA of low as 30 percent may have been considered *highly sensitized* prior to the widespread use of kidney paired donation (KPD) programs and prioritization of sensitized patients in deceased donor allocation schemes. In the current era, it is best to avoid terms such as *highly sensitized* and instead report the cPRA and mode of sensitization which is more informative in terms of a patient’s allocation priority, probability of receiving an organ offer, and risk of antibody mediated rejection (ABMR).

## Who Derives the Most Benefit from Desensitization in the Current Era?

Desensitization protocols are generally used for the following two reasons: 1) to increase transplant candidates’ access to transplantation by decreasing HLA antibody and the number of unacceptable antigens for listing (e.g. reduction in cPRA), or 2) to decrease known DSA prior to a planned positive crossmatch transplant to reduce the risk of immediate graft loss from catastrophic hyperacute rejection. The term desensitization has often been used loosely to refer to any treatment given in the context of known donor specific antibody or positive crossmatch even if the treatment was not expected to decrease alloantibody (e.g. complement inhibitors).

In the last two decades, the need for desensitization or HLA incompatible transplantation has evolved. Before widespread kidney paired donation and changes in donor allocation schemes to prioritize sensitized patients, there was a great need for effective desensitization therapy and positive crossmatch transplantation because of the increasing number of sensitized patients on the waiting list with prolonged waiting times and disproportionately low rates of transplantation particularly for patients with a cPRA > 95% (5). In an attempt to overcome the humeral barriers to transplantation aggressive desensitization strategies were employed. While these treatments did allow some patients to get transplanted, their effectiveness was variable and graft failure from long term chronic active ABMR was common (6, 7).

In the background of aggressive desensitization strategies came a rising in the utility of KPD (8, 9). Kidney Paired Donation was first introduced in the late 1980s in South Korea (10), but did not gain traction in the United States until 2007 when the legality of this practice was clarified with the Charlie Norwood Living Organ Donation Act. Since that time, kidney paired donation has become widespread. In 2019, over 1100 living donor kidney transplants were facilitated through KPD constituting about 16% of all living donor transplants (11). While there was initial enthusiasm that kidney paired donation would eliminate the need for desensitization and positive crossmatch transplant, it was soon realized that KPD was not sufficient and KPD pools became saturated with sensitized patients (12). Although these programs increase the number of potential donors for sensitized individuals, patients with antibodies against a wide variety of HLA antigens may still not be able to find a crossmatch-negative donor, even if the donor pool is very large. Data from the 3-Mayo site kidney paired donation program found that having a cPRA of > 98% was a risk factor for waiting in kidney paired donation greater than 3 months (13). Data from the National Kidney Registry is comparable (12). Because some sensitized patients may not be able to find an HLA compatible donor, many programs combine KPD with positive crossmatch transplantation to find a donor with a more favorable crossmatch and DSA profile (14, 15).

**Figure 1:**
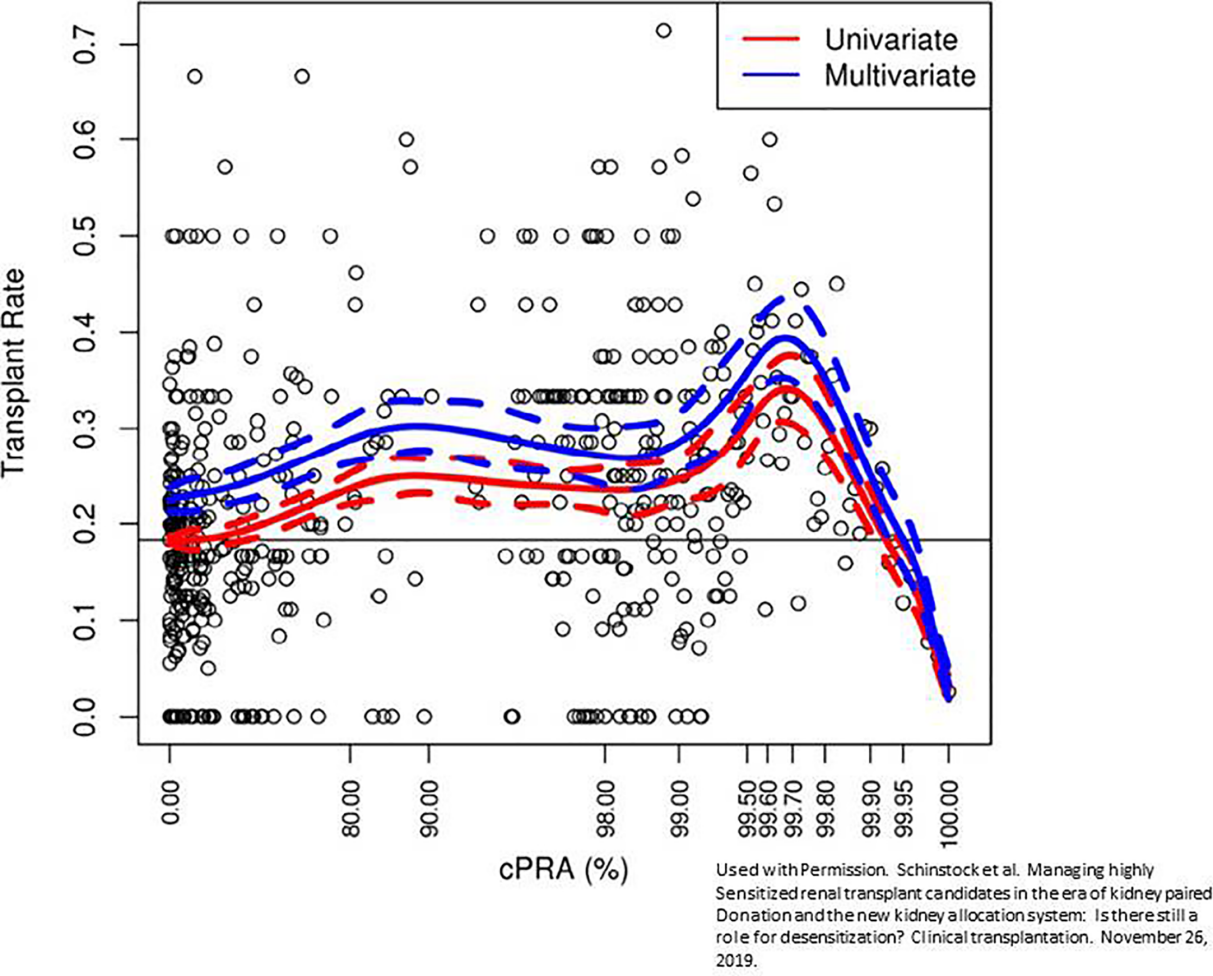
Reduced transplantation rate among patients with cPRA > 99.9%. Multivariate fit of transplantation rate versus calculated panel reactive antibody (CPRA). A fit of transplant rate versus cPRA using a restricted cubic spline with 95% confidence interval controlling for time on the waitlist, age, gender, blood type, waitlist region, and ethnicity. The red line is univariate, while the blue line indicates the multivariate fit corresponding to a candidate with male gender, blood type A, waitlist region 5, Caucasian ethnicity, and waiting time of 2.5 y. Markers represent the observed transplant rate within each window of CPRA of width 0.01%. This prevalent cohort was active as of June 1, 2016, and followed for change in status through June 1, 2017. Used with permission. Schinstock et al. Managing highly sensitized renal transplant candidates in the era of kidney paired donation and the new kidney allocation system: Is there still a role for desensitization? Clinical transplantation. November 26, 2019 (19).

Given the realization that desensitization and KPD were ineffective strategies for getting many sensitized patients to transplant, deceased donor allocation schemes were revised. In December of 2014, a new kidney allocation system came into effect in the United States that gave tremendous priority to the sensitized patient (16, 17). In the system used previously, all candidates with a cPRA of >80% were given an additional 4 allocation points. Currently, additional allocation points are given on a sliding scale starting with a cPRA as low as 20%. Major priority is given to candidates with a cPRA ≥98%. Candidates with a cPRA of 98%, 99%, and 100% get an additional 24.4, 50.09, and 202.1 additional allocation points, respectively (17). Within months of the implementation of this allocation system, hundreds of transplant patients with cPRA ranging from 90-100% were fortunate to receive a deceased donor transplant after years of waiting (18). However, even with these allocation changes, patients with a cPRA of > than 99.9% continue to have very low rates of kidney transplantation **Figure 1** (19). Candidates with this degree of sensitization are not rare. 70% of patients with a cPRA of 100% on the UNOS waiting list have a cPRA > 99.9% (19). Not only are these candidates disadvantaged from a transplant perspective, they are at a higher risk for waitlist mortality (20, 21). The problem is that there are simply not enough organs available with unique HLA antigens to be compatible with these candidates, thus further expansion in KPD or changes in allocation policy will not adequately solve this problem (22). Even a small decrease in cPRA among these highly sensitized patients with desensitization has the potential to markedly improve access to transplantation.

It is also worth mentioning that as sensitization increases, the rate of living kidney donor transplantation decreases (19). Analysis of UNOS data has shown that approximately 26% of patients with a cPRA of less than 80% received a living donor kidney transplant compared to 6.5% of candidates with a cPRA > 80% (19). Furthermore, only 2.5% of candidates with 100% cPRA received a living donor transplant and only about half of these were facilitated through KPD (19). These data suggest that there may be a role for desensitization in select cases to facilitate living kidney transplantation given the clear benefits of living versus deceased donor kidney transplantation.

Although the needs for desensitization in kidney transplant have changed in the last two decades, it remains clear that a select group of transplant candidates could derive benefit from effective desensitization. Kidney transplant candidates with a cPRA of > 99.9% have the greatest need for desensitization. One could also argue that select patients with a cPRA < 98% with a incompatible approved living donor or who have been active on the waiting list for several years may also derive benefit from desensitization considering the patient survival benefit of getting off of dialysis (23, 24). These are patients who should be targeted for clinical trials **Table 1**.

**Table 1:** Kidney transplant candidates who may benefit from Desensitization.

1. All kidney transplant candidates with cPRA > 99.9%. These candidates have reduced transplantation rates on the deceased donor list and less likely to benefit from kidney paired donation.
2. Kidney transplant candidates with cPRA > 98% and > 5 years of waiting time. These are candidates who have not benefited from their allocation priority.
3. Kidney transplant candidates with an approved living donor and cPRA >98% but have not had a compatible offer through kidney paired donation.

## Kidney Transplant Outcomes Following Desensitization

In addition to understanding *who* would most benefit from desensitization, it is key to understand the outcomes following transplant with DSA to make personalized clinical decisions weighing the risks of incompatible transplantation versus remaining on the waiting list. Despite the patient survival advantage of HLA incompatible transplant; HLA incompatible transplants are associated with reduced allograft survival (6, 25), increased expense (26), and increased hospital readmission rates (26). Understanding the immunological risk specific to a particular donor/recipient pair is a core principle in the transplantation of sensitized patients. In general, the quantity of antibody at the time of transplant correlates with risk, and the level of antibody can be semi-quantitatively determined with a combination of a variety of tests including the solid phase assay SAB mean fluorescence intensity (MFI), titers, cytotoxic and flow cytometric crossmatches, and C1q antibody positivity (27).

In the modern era, kidney transplantation is rarely performed in the setting of high levels of DSA as defined by a positive cytotoxic crossmatch and DSA MFI > 10,000 to avoid immediate allograft loss from hyperacute rejection. In patients with pre-transplant positive cytotoxic crossmatch, the risk of hyperacute rejection and aggressive early ABMR can be as high as 20% and 70% respectively. By 1 year, nearly 50% of those grafts fail (28).

The outcomes after HLA incompatible kidney transplantation with negative cytotoxic crossmatch varies and is largely based on single center retrospective studies with heterogeneous immunosuppression and desensitization protocols. The reported incidence of early acute active ABMR associated with the memory response is as low as 1% among kidney transplant recipients with DSA based on single antigen bead positivity only to up to nearly 40% among patients with DSA positivity based single antigen beads and a high positive flow cytometric crossmatch (7, 29–32). In a large retrospective series among a French cohort, the incidence of early active ABMR was 36.4% with a baseline DSA MFI of 3001-6000 and 51.3% with a baseline DSA MFI of > 6000^7^. A key message is that transplantation in the context of low level DSA results in acceptable outcomes when other options are not available.

Even when early allograft loss within the first year post-transplant is avoided, chronic active ABMR remains a major problem. At 5 years post-transplant over 50% of surveillance biopsies have features of chronic active ABMR among patients transplanted with DSA and a high positive B cell flow cytometric crossmatch (defined as mean channel shift of 250 with < 106 considered negative). Renal allograft survival following incompatible transplantation also correlates with the amount of DSA at the time of transplantation (25, 30, 33). A multicenter observational study of living donor transplants performed at 22 centers in the United States showed that the 1 and 5 year unadjusted all-cause graft loss was 3.9% and 16.6% among patients without DSA at transplant, 3.8% and 20.2% when SAB were positive for DSA but the flow crossmatch was negative, 6.9% and 28.8% when the flow cytometric crossmatch was positive, and 19.4% and 39.9% when the cytotoxic crossmatch was positive (25).

## Emerging Therapeutics for Desensitization

Unfortunately, desensitization studies are retrospective single center experiences that include heterogeneous candidates with varied levels of baseline sensitization and a lack of standard endpoints, thus it is difficult to compare the efficacy of the various protocols. Most desensitization protocols include plasmapheresis to reduce circulating HLA antibody and intravenous immunoglobulin for its immunomodulatory effects and to prevent hypogammaglobinemia, but many other therapies have been added or used alone **Table 2**.

**Table 2:** Desensitization therapies in kidney transplantation.

Drug class	Name	Mechanism of Action	Previous and ongoing studies	Key Features
Plasmapheresis	NA	Removal of circulating immunoglobulin	Stegall et al. (28)	
Intravenous Immunoglobulin	NA	Exact mechanism unknown. Multiple Immunomodulatory mechanisms.	Glotz et al. (34) Jordan et al. (35) Stegall et al. (28)	
Anti-CD 20 monoclonal antibodies	Rituximab	Depletes B cells	Jordan et al. (36) Vo et al. (31) Jackson et al. (37)	
	Obinutuzumab		Redfield et al. (38)	3^rd^ generation anti-CD20 dependent on ADCC. Used in for relapsed hematologic malignancies.
Proteosome inhibitors	Bortezomib	Accumulation of unwanted cellular protein and apoptosis.	Woodle et al. (39) Moreno Gonzalez et al. (40)	Reversible proteasome inhibitor
	Carfilzomib		Tremblay et al. (41)	Irreversible proteasome inhibitor. Less neurotoxicity than bortezomib.
	Ixazomib		Ongoing ClinicalTrials.gov Identifier: NCT03213158	First oral proteasome inhibitor
Anti-CD38 monoclonal antibodies	Daratumumab	Depletes plasma cells	Kwun et al. (42)	Studied in nonhuman primate model and was associated with increased in T cell mediated rejection.
	Isatuximab		Ongoing ClinicalTrials.gov Identifier: NCT04294459	
Cysteine protease	Imlifidase	Cleaves heavy chains of human IgG (all subclasses) and eliminates IgG effector functions	Jordan et al. (43) Jordan et al. (44)	Rebound of DSA at Day 7. Retreatment with imlifidase often ineffective because of the development of neutralizing antibodies.
Interleukin-6 Blockade	Tocilizumab	IL-6 receptor inhibitor	Vo et al. (45)	
Complement inhibitors*	Eculizumab	Terminal complement blockade to protect against antibody mediated rejection.	Stegall et al. (46) Marks et al. (47) Glotz et al. (48)	

*Does not deplete antibody and therefore not a “desensitization” agent.

### Anti-CD 20 Monoclonal Antibodies

Many desensitization regimens also include the chimeric anti-CD 20 antibody rituximab aimed to deplete B cells and minimize the memory response (31, 36, 49, 50). Rituximab has been shown to reduce the PRA, increase the rate of transplantation, and decrease the pretransplant flow cytometric crossmatch mean channel shift (50); but even after treatment, nearly 50% of patients had ABMR within 30 days post-transplant (50). More recently Obinutuzumab has been studied in desensitization (38). This 3^rd^ generation anti CD 20 monoclonal antibody has been associated with a more profound depletion of B cells and is used outside of transplant as a second line agent for hematologic malignancies refractory to rituximab. Similar to rituximab, Obinutuzumab is associated with depletion of peripheral and lymph node B cells, but its effect on MFI, number of unacceptable antigens, and cPRA has been shown to be limited and does not appear to be clinically meaningful (38).

### Proteasome Inhibitors

Many therapies that have been studied in desensitization were first used in multiple myeloma because long lived CD38 positive plasma cells that reside in the bone marrow and constitutively secrete alloantibody are the target for both indications. Bortezomib, a reversible proteasome inhibitor, has been shown in *in vitro* models to deplete bone marrow derived plasma cells (51). In clinical studies, this therapy led to modest reductions in alloantibody, but was not well tolerated (39, 40). Similarly, the irreversible proteasome inhibitor carfilzomib has been shown to deplete plasma cells and decrease HLA antibody, but its effects were transient and antibody levels returned to baseline in less than 6 months (41).

### Anti-CD38 Monoclonal Antibodies

Daratumumab, an anti CD38 monoclonal antibody, has been studied for desensitization in a nonhuman primate model. This treatment was associated with reduced DSA and prolonged renal survival but was also followed by a rebound in DSA and a severe combined antibody and T cell mediated rejection leading to graft loss (42). The concomitant T cell mediated rejection was likely from depleting regulatory cell populations (42). Daratumumab has also been used to successfully desensitize a heart transplant candidate. The cPRA dropped from 80% to 62% after daratumumab and the heart candidate received an organ with HLA that included two antigens that were considered unacceptable before treatment (42). A similar but more potent anti CD38 monoclonal antibody, isatuximab, is currently being studied in a phase 1b/2 trial to evaluate the safety, pharmacokinetics, and efficacy for desensitization in kidney transplant candidates [ClinicalTrials.gov Identifier: NCT04294459].

### Interleukin-6 Blockade

Interleukin 6 is a proinflammatory cytokine that is central to the acute inflammatory response. It has multiple roles in mediating innate and adaptive immune responses including activation of T helper 17 cells and inhibiting regulatory T cells. Particularly relevant to sensitization, IL-6 is critical for maintaining long lived plasma cells in their niche. A small phase I/II nonrandomized study of the IL-6 inhibitor tocilizumab was used for desensitization among 8 patients who were considered refractory to rituximab and IVIG (45). The use of tocilizumab was associated with reduction in an immunodominant DSA score based on MFI (45). Further studies of IL-6 inhibitors have not been published for pretransplant desensitization; but a randomized multicenter randomized clinical trial using clazakizumab, a soluble IL-6 inhibitor, for chronic active ABMR is currently enrolling patients [ClinicalTrials.gov Identifier: NCT03744910].

### Cysteine Protease

A novel agent that has shown promise in desensitization is Imlifidase. This endopeptidase rapidly cleaves all IgG into F(ab’) and Fc fragments to impair the effector function from all circulating IgG. In both phase 1 and 2 desensitization trials, this agent led to a precipitous drop in DSA within hours, and therefore is a valuable tool for deceased donor positive crossmatch transplantation to avoid hyperacute rejection (44, 52). The main limitation is that this drug will likely need to be part of a combination therapy regimen because it only cleaves circulating antibody and antibody levels begin to have a brisk rebound within 3-7 days (44, 52). In the future, it may be used instead of pretransplant plasmapheresis to rapidly reduce circulating DSA.

### Complement Inhibitors

Eculizumab is a terminal complement inhibitor that does not decrease antibody but has been added to desensitization regimens to minimize the effect of a high level of DSA on the allograft. It has been shown to decrease the incidence of early active ABMR in a small single center cohort from nearly 41.2% in the historical control group compared to 7.7% in the treatment arm (46). Larger multicenter studies in living and deceased donor populations have not confirmed these results, but also included patients at lower risk for ABMR at baseline (47, 48). Regardless, eculizumab has not been shown to improve long term allograft survival when added to desensitization (53, 54). While the long term studies of terminal complement blockade have been disappointing, there may be a role for adding eculizumab to novel high-risk desensitization protocols in the future to minimize the risk of early allograft failure.

## Novel Approaches to Clinical Trial Design in Desensitization

### Use of cPRA and Antibody Titer for Trial Endpoints

Recurrent themes in the desensitization field are the small heterogeneous study populations with varied baseline sensitization, differing access to living donors, and unique desensitization endpoints. The heterogeneous endpoints range from the change in MFI of HLA antibody based on SAB results to the rate of transplantation. Often the endpoints used are subject to laboratory variability and differences in center transplantation practices. Standardizing endpoints and requiring minimal standards for laboratory reporting would be a major advancement in this field.

Of the various endpoints for desensitization, cPRA is advantageous because it can be applied to candidates with and without a living donor and eliminates the bias that occur from varied deceased donor acceptance, availability of a living donor, or access to kidney paired donation. It is easy to measure and directly related to a candidate’s probability of receiving a kidney transplant but could be also used in heart and/or lung desensitization studies. The main drawback of cPRA is that is often based on the MFI from undiluted serum samples, and it is well known that MFI results are impacted by inherent assay limitations and intra-laboratory variability particularly in the sensitized patient. Another weakness is that the cPRA can be an insensitive measure of desensitization if the antibody is not decreased enough to change the number of unacceptable antigens. Stepwise dilution of the serum will gradually eliminate antibody positivity and decrease the cPRA, thus determining the *cPRA per titer* can overcome these limitations.

**Figure 2:**
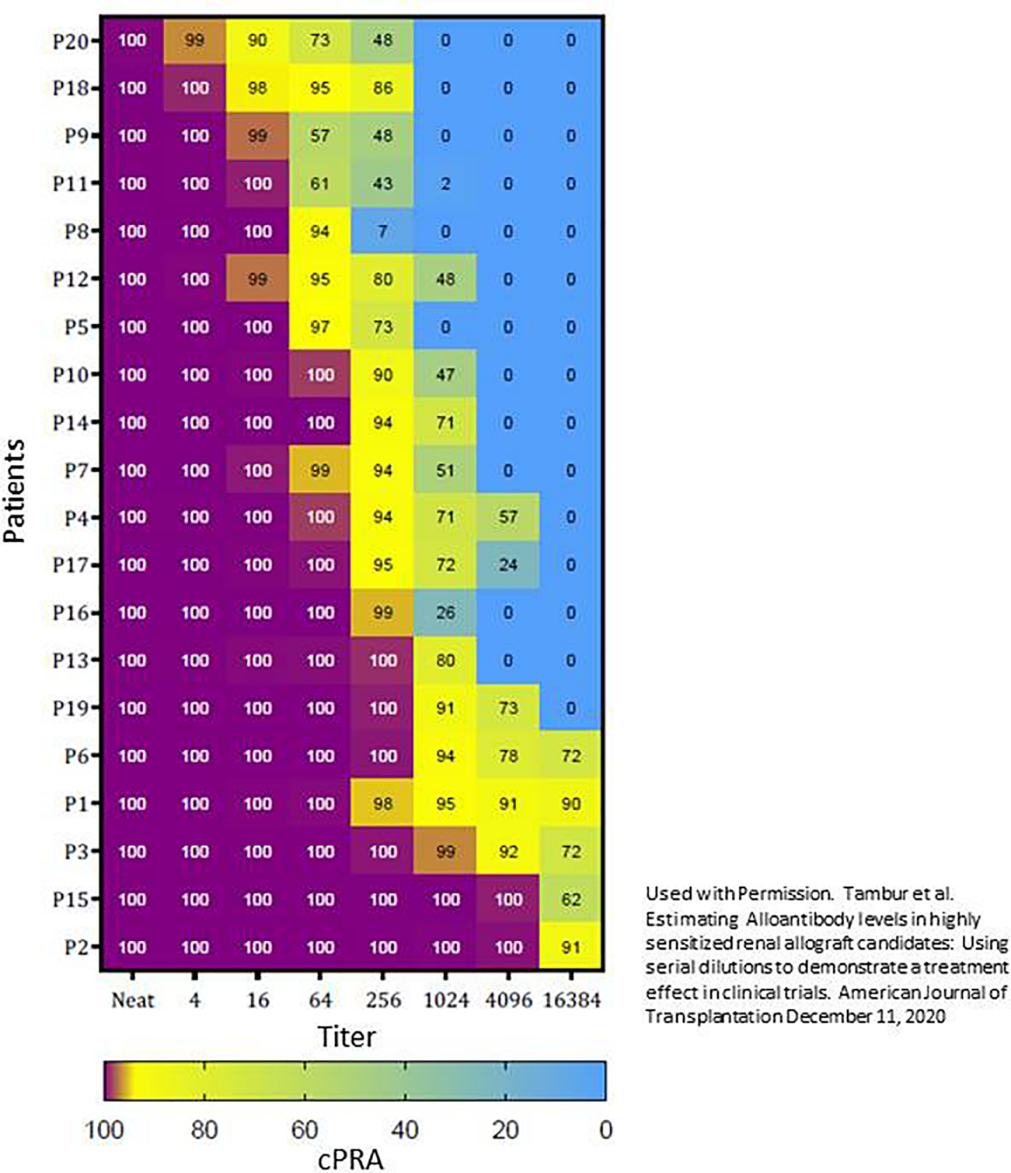
Using titer to stratify patients with cPRA > 99.9%. This stratification was based on the first replicate from the baseline sample. The heat map shows the cPRA obtained from positive antibody specificities per titer for each of the 20 patients. The patients were ordered to show patients who would be the most likely to respond to desensitization (top) to least likely to respond to desensitization (bottom). For example, P2 continued to have a 100% cPRA when serum was diluted 1:4096, and thus it may be extremely difficult to remove enough antibodies to render this patient transplantable. Used with Permission. Tambur et al. Estimating Alloantibody levels in highly sensitized renal allograft candidates: Using serial dilutions to demonstrate a treatment effect in clinical trials. American Journal of Transplantation December 11, 2020 (55).

We evaluated cPRA reduction per titer among 20 sensitized patients with a cPRA > 99.9% **Figure 2**. We found that titer determination in a central laboratory using same lot reagents and batch testing leads to reproducible results and that the cPRA per dilution remains constant within approximately 1 titer if serum samples are obtained within 1 year (55). Transplant candidates with a cPRA of > 99.9% have vastly different quantities of antibody (55). While the cPRA began to drop after only 1-2 dilutions for some candidates, other candidates remained at cPRA >99.9% even when their serum was diluted 1:4096. Presumably those candidates whose cPRA decreased with fewer dilutions would be easier to desensitize, thus cPRA per titer could also be used to develop the inclusion criteria for a clinical trial or risk stratification.

The concept of determining the antibody titer has applications beyond the clinical trial including the appropriate assignment of unacceptable antigens, evaluating the efficacy of desensitization, quantifying the change in DSA post-transplant, or evaluating the response to ABMR therapies. We recognize the time and expense to do serum dilutions and titers, but it is important to recognize that multiple serial dilutions are only rarely needed. The number of dilutions tested truly depends on the purpose of testing. For example, if you are willing to attempt desensitization therapy if the antibody is < 1:8, you may only test a 1:8 dilution. In other cases, you may choose to start with testing one dilution (e.g. 1:64) and decide on further testing based on those results. The key is to acknowledge the limitations of the MFI and know when and how to use the titer measurement in practice.

### Novel Clinical Trial Designs

With the expansion of the therapeutic options now available for desensitization combined with the probable need for combination therapy comes the opportunity to adapt new strategies for evaluating the safety and efficacy of desensitization protocols. It is simply not feasible to conduct multiple randomized controlled trials to efficiently evaluate novel desensitization strategies. Resources and patients eligible to participate in these trials are finite. Novel adaptive trial designs can be utilized to efficiently study small heterogeneous populations with combination therapeutic regimens and address issues with suboptimal enrollment (56). Adaptive clinical trials adapt depending on predefined outcomes generally based on Bayesian probabilities. The goal of these designs is to learn quickly what does or does not work and halt the study of a therapeutic agent or combination early if futile or unsafe. These designs are most efficient if validated surrogate endpoints such as cPRA are used.

**Figure 3:**
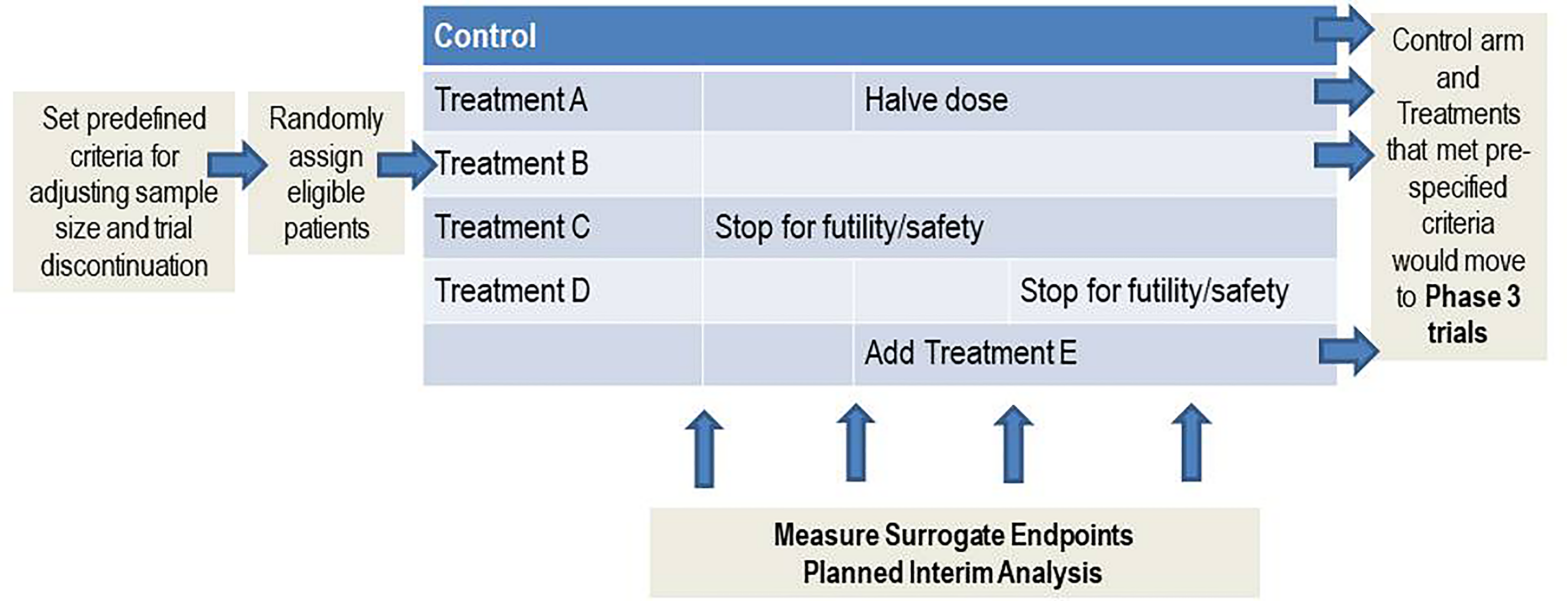
Platform master protocol design. A *platform* master protocol allows for the study of multiple therapies for a single disease perpetually, and therapies can enter or leave the trial of the basis of predefined criteria. This master protocol is ideal for studying small heterogeneous populations.

The foundation of these designs is a master protocol (57). Examples of master protocols include *umbrella, basket*, and *platform*. *Umbrella* master protocols are used to study multiple targeted therapies for a single disease while a *basket* protocol would be used to study a single therapy for multiple diseases. A *platform* master protocol is essentially an extension of the umbrella design, but multiple therapies are studied for a single disease perpetually and therapies can enter or leave the trial of the basis of predefined criteria **Figure 3**. The control arm and therapies that meet pre-specified criteria can move onto a phase 3 clinical trial. This platform design would be ideal for desensitization because of the relatively small patient population and multiple different desensitization combinations that need rigorous study. In fact, sensitized kidney, heart, and lung candidates could be studied in the same trial if an endpoint such as cPRA was used.

## Conclusion

In summary, there remains an unmet need for desensitization for candidates with the highest degree of sensitization who have not benefited from KPD or organ allocation policy changes. Many new therapeutic options are available, and we are hopeful that the use of new endpoints and clinical trial designs in this field will lead to effective desensitization approaches in the future to increase access to transplantation to patients in the most need.

## Author Contributions 

CS, AT, and MS all contributed equally in the design of the manuscript, literature review, writing of manuscript, and revision of manuscript. All authors contributed to the article and approved the submitted version.

## Conflict of Interest

CS and MS have research contracts with Sanofi and CSL Bering and prior research contracts with Alexion pharmaceuticals. AT has received reagents and supplies for titer studies as a gift from One Lambda/A Thermo Fisher Brand.

## Abbreviations

DSA, donor specific antibody; HLA, human leukocyte antigen; PRA, panel reactive antibody; SAB, single antigen bead; cPRA, calculated panel reactive antibody; KPD, kidney paired donation; ABMR, antibody mediated rejection; MFI, mean fluorescence intensity.
